# Perspectives on Converting Keratin-Containing Wastes Into Biofertilizers for Sustainable Agriculture

**DOI:** 10.3389/fmicb.2022.918262

**Published:** 2022-06-20

**Authors:** Qingxin Li

**Affiliations:** Guangdong Provincial Engineering Laboratory of Biomass High Value Utilization, Institute of Biological and Medical Engineering, Guangdong Academy of Sciences, Guangzhou, China

**Keywords:** keratin, feather degradation, fertilizers, protein, value-added chemicals, keratinase

## Abstract

Keratin-containing wastes become pollution to the environment if they are not treated properly. On the other hand, these wastes can be converted into value-added products applicable to many fields. Organic fertilizers and biofertilizers are important for sustainable agriculture by providing nutrients to enhance the growth speed of the plant and production. Keratin-containing wastes, therefore, will be an important resource to produce organic fertilizers. Many microorganisms exhibit capabilities to degrade keratins making them attractive to convert keratin-containing wastes into valuable products. In this review, the progress in microbial degradation of keratins is summarized. In addition, perspectives in converting keratin into bio- and organic fertilizers for agriculture are described. With proper treatment, feather wastes which are rich in keratin can be converted into high-value fertilizers to serve as nutrients for plants, reduce environmental pressure and improve the quality of the soil for sustainable agriculture.

## Introduction

**Figure 1:**
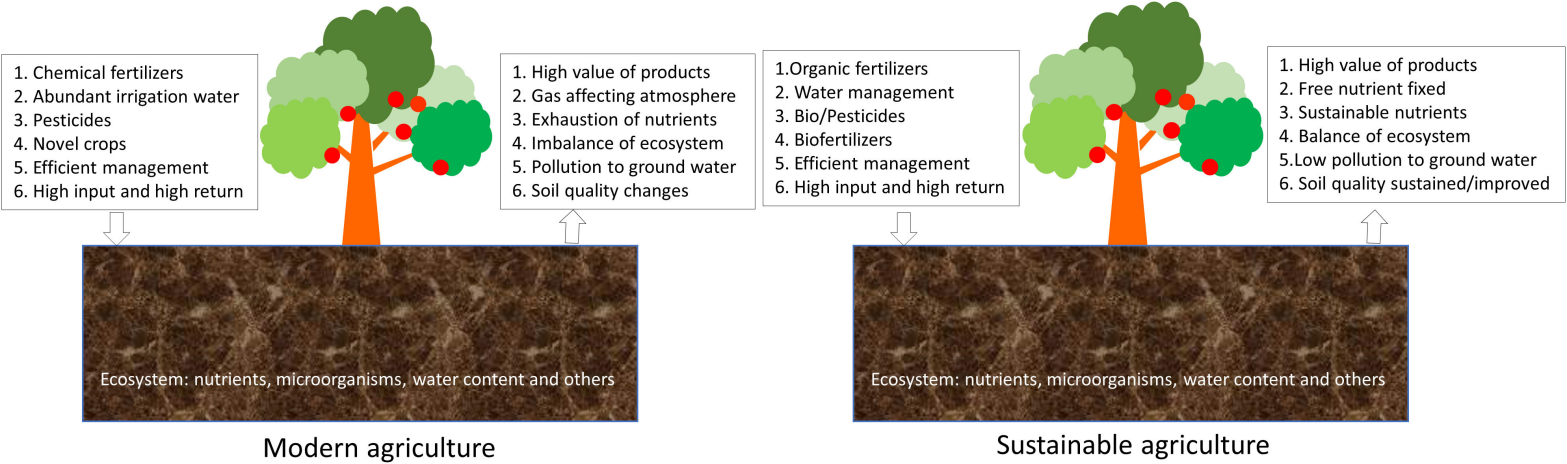
Modern agriculture and sustainable agriculture. The difference of these two types of agriculture is included.

Modern agriculture can be considered as farming with improved technologies and strategies to increase food production and reduce the consumption of resources and energy. It brings in large quantities of high-quality and safe food to support and improve everyday life (Singh et al., 2021; Ranjha et al., 2022). The impact of modern agriculture on the environment is of great concern due to its effect on the soil, water, and our living environment (Figure 1). The high efficiency of food products results in loss of nutrients in the soil, which in turn will affect the yields of the crops in the future. The application of fertilizers and other chemicals such as pesticides will contaminate soil and groundwater, giving rise to threats to the environment (Humbert et al., 2016; Vejan et al., 2021; Martins-Gomes et al., 2022). Therefore, it is necessary to reduce the environmental pressure and recover the soil nutrient using suitable strategies (Zhuang et al., 2019; Tan et al., 2022). Sustainable agriculture was proposed to provide high-quality food for human beings without damaging the natural environment (Figure 1). Technologies, novel agriculture-related products, and strategies are explored to meet the requirement of sustainable agriculture by reducing soil erosion, relieving contamination of groundwater due to the application of artificial chemicals and excess amounts of fertilizers and recovering the eco-system by reducing the damages to the environment through bioremediation and applications of suitable fertilizers (Raliya et al., 2018; Ali et al., 2021; Rajput et al., 2021).

### Fertilizers for Agriculture

**Figure 2:**
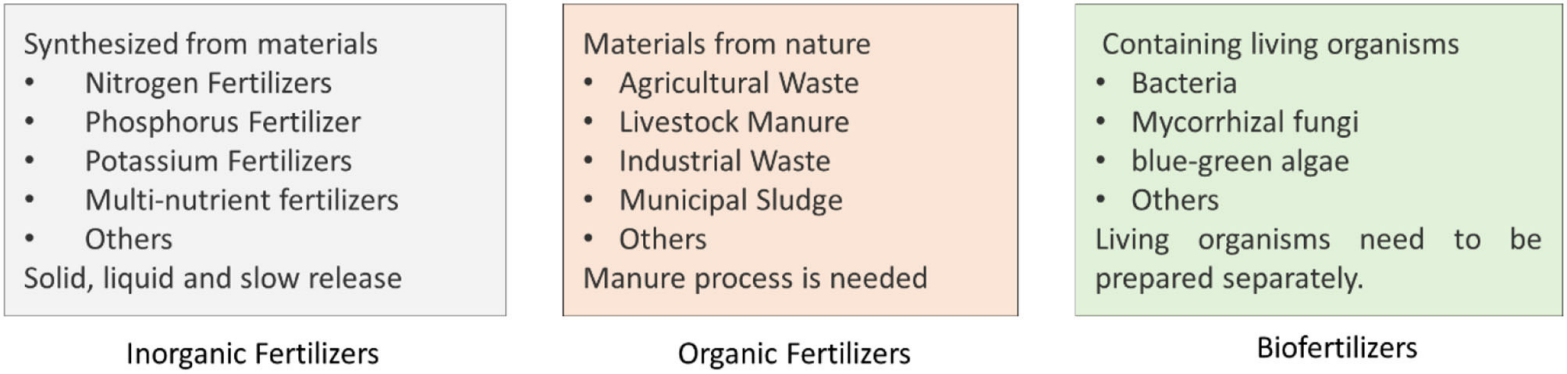
Three types of fertilizers. Three types of fertilizers based on different criteria are shown. Biofertilizers will be playing important roles in sustainable agriculture due to the advantages to sustain the ecosystem of soil and improving growth of the plants.

Fertilizers are usually rich in nutrients such as nitrogen, phosphorus, and potassium and can be prepared through different strategies (Figure 2) (Nazir et al., 2021; Ngo et al., 2021). Fertilizers can be classified into different types based on their components such as mineral fertilizers and methods in manufacture such as organic, inorganic fertilizers, and biofertilizers (Hirel et al., 2011; Liu et al., 2021; Urmi et al., 2022). The application of fertilizer improves the life of human beings while it also causes environmental problems (Herridge et al., 2008; Hirel et al., 2011; Zilio et al., 2022). Chemically synthesized fertilizers have been widely used in agriculture because of their high purity, easy application, and fast absorption by the plants (Hirel et al., 2011). With the wide application of fertilizers, their impact on the ecosystem such as atmosphere, soil microorganisms, and groundwater in soil has been highly recognized (Liu et al., 2021; Guo et al., 2022; Lisowska et al., 2022; Maeda, 2022). For example, the application of synthesized nitrogen-containing fertilizer can supply the nutrient for fast growth of crops while excess amount of nitrogen can be converted into nitrogen gas which can be released to the atmosphere to serve as one of the greenhouse gases causing global warming (Gao et al., 2022; Islam et al., 2022; Mikhael et al., 2022). Unutilized components from the fertilizers can be released into the groundwater to cause contamination. Therefore, suitable strategies are required to remove the contamination in the groundwater (Burow et al., 2010; Petzet and Cornel, 2011; Gu et al., 2013; Harris et al., 2022).

### Biofertilizers in Agriculture

To avoid the potential threats caused by the overuse of chemical fertilizers to the ecosystem, organic fertilizers and biofertilizers have been recognized as an important element in sustainable agriculture (Abdel-Hamid et al., 2021; Kumar et al., 2022b; Yang et al., 2022). The combination of these fertilizers with chemical fertilizers can provide nutrients for plants and reduce the threat of chemical fertilizers (Kumar et al., 2022b). At the same time, these fertilizers can improve the environment of soil and solubilize the elements such as nitrogen and phosphate in soil to avoid loss of nutrients and contamination of the environment (Nosheen et al., 2022). The addition of microorganisms in the fertilizers will be very useful for balancing soil microorganisms, which will also be critical for plant growth and sustaining the ecosystem (Young and Ritz, 2000; van der Heijden et al., 2008; Saleem et al., 2019). Although both organic fertilizers and biofertilizers can be produced from animal and plant sources, biofertilizers contain microorganisms important for plant growth or the soil environment. Despite their differences, organic fertilizers can be mixed with biofertilizers to increase their efficiency (Al-Amri, 2021). Therefore, it is an economic way to produce biofertilizers using animal or plant wastes that are rich in carbon, nitrogen, and other elements. The resulting biofertilizer will contain both nutrients for the growth of plants and microorganisms that can be helpful for the ecosystem of soil. Keratin is a fibrous protein and presents in many wastes such as feathers from the poultry industry, representing a class of resource that can be converted into value-added chemicals. Feathers contain over 90% keratin and occupy 5–7% total weight of chicken (Adav et al., 2018; Li, 2019). Feathers have become one of the pollutants to the environment if they are not properly processed. Feathers can be converted into different materials used in different fields (Barone et al., 2005; Dias et al., 2010; Li, 2019; Tamreihao et al., 2019).

In this review, the application of feather-a keratin-containing waste in the generation of biofertilizers for sustainable agriculture is described. Feathers from the poultry industry will serve as a low-cost resource for producing fertilizers for agriculture, which overcomes the challenges in modern agriculture. With the inclusion of certain microorganisms, feather keratin can be converted into biofertilizers which contain nutrients for plant growth and microorganisms important for the soil ecosystem.

## Keratin

### Structure and Composition of Keratin

Keratin is a class of protein and presents in feathers, hair, hooves, wool, and nails (McKittrick et al., 2012; Chilakamarry et al., 2021). Keratin is a highly stable protein because of its high content of cysteine residues to form disulfide bonds and the presence of hydrogen bonds and hydrophobic interactions to stabilize its structure (Vidmar and Vodovnik, 2018). Fibrous keratin is not soluble even in some organic solvents, water, diluted acids, and alkaline solutions, making them resistant to normal proteases such as pepsin and trypsin (Vidmar and Vodovnik, 2018). Keratin can be classified into α-keratin and β-keratin according to the secondary structures of the protein chains (Fraser and Parry, 2008, 2011; Lange et al., 2016). The types and contents of amino acids affect the structures and properties of keratin (Qiu et al., 2020). The polypeptide chains in keratin are packed into a more complicated structure through inter- and intra-residue disulfide bonds *via* cysteine residues, hydrogen bonds, and hydrophobic interactions (Vidmar and Vodovnik, 2018). Based on the content of disulfide bonds, keratin is classified into soft and hard keratins. Feathers are hard keratins, and the main component of its keratins is mainly β-keratins. It has been noted that feathers also contain both α- and β-keratins. Like some proteins in human bodies, keratin can also be modified after translation such as phosphorylation, sumoylation, and glycosylation. Modification of keratin can play a role to affect its structure and stability (Snider and Omary, 2014). Like cellulose, lignin and hemicellulose are abundant in nature, keratin represents a class of renewable organic polymers (Lange et al., 2016; Bealer et al., 2020) and represents a valuable source for providing nutrients for both animals and plants (Pettett et al., 2004; Gurav and Jadhav, 2013; Bhari et al., 2021).

### Keratin Waste-Feather

Feathers are one of the common byproducts in poultry industry. Due to increase of human population and higher demand for chicken-derived food, the yield of feather is increasing annually (Verma et al., 2017; da Silva, 2018). It is estimated that over 2 million tons of feathers generated from the poultry industry globally and proper treatment is needed to handle this byproduct to avoid generation of toxic reagents to the environment (Tamreihao et al., 2019). Feathers can be processed into some starting materials using different strategies to serve as a starting material, which will eliminate their impact on the environment. As feathers from poultry industry are always mixed with water and other wastes (Tamreihao et al., 2019), they provide an environment for the growth of many pathogenic microorganisms or contain some toxic compounds to the environment (Franke-Whittle and Insam, 2013; Dréano et al., 2021). In addition, the presence of microorganisms in feather waste could result in the releasing of pollutants such as nitrous oxide, ammonia, and hydrogen sulfide that can be released into the atmosphere (Tesfaye et al., 2017). Physical processing such as incineration and controlled landfilling will result in waste of such a large amount of organic substance (Tesfaye et al., 2017). Another way to treat feathers is to convert them as a material for making clothes, decorations, medical devices, fertilizers, dusters, bedding materials, and feedstocks using a fast and simple procedure such as washing and chemical treatment (Papadopoulos et al., 1986). Feathers can be converted into animal feeds through treatment with chemicals and steam pressure cooking method, but all these processes require a large amount of energy and the nutrients in feathers are not well maintained (Papadopoulos, 1989; Latshaw et al., 1994; Wang and Parsons, 1997). Converting feather waste into value-added products beneficial to the society using an economic strategy is of great interest to many researchers (Kang et al., 2018; Jain et al., 2016). Although chemical hydrolysis of feathers can produce soluble proteins and amino acids, it requires significant energies and impacts the quality of the product such as proteins and amino acids (Kim et al., 2001; Joardar and Rahman, 2018; Li, 2019). Accumulated studies have shown that microbial degradation of feathers is feasible and quite a number of microorganisms that are able to degrade keratin or keratin-containing wastes have been identified (Calin et al., 2017; Qu et al., 2018; Vidmar and Vodovnik, 2018; Bohacz and Korniłłowicz-Kowalska, 2019). The available studies suggested that it is feasible to convert feathers into value-added products such as biofertilizers through microorganisms, which provides a strategy to make full use of keratin from these wastes and generate low-cost products for sustainable agriculture (Tamreihao et al., 2019).

## Microbial Degradation of Keratin

### Keratin Degradation by Microorganisms

Keratin is resistant to normal proteases and not soluble in water while there is no accumulation of keratin wastes in nature, which is due to the contribution of microorganisms (Williams and Shih, 1989). Indeed, bacteria, actinomycetes, and fungi are shown to have the capability to degrade keratin (Calin et al., 2017; Bohacz and Korniłłowicz-Kowalska, 2019). Many researchers have isolated keratin-degrading microorganisms from different environments and proved that these microorganisms can be utilized to convert keratinous wastes into value-added products (de Menezes et al., 2021; Nnolim and Nwodo, 2021). These isolated microorganisms exhibited their potential to degrade keratin and keratin-containing wastes such as feathers into soluble fractions and amino acids (Callegaro et al., 2018; Shanmugasundaram et al., 2018; Bohacz, 2019; Tamreihao et al., 2019; Chaudhary et al., 2021). Fermentation conditions have been explored to obtain better keratin degradation efficiency (Rajput and Gupta, 2013). Both submerged and solid-state fermentation conditions were investigated to explore the optimal conditions for keratin degradation (De Azeredo et al., 2006). Mutagenesis induced by various ways has been explored to improve the efficiency. For example, ethyl methanesulfonate was applied to a bacterium *Bacillus subtilis* LFB-FIOCRUZ 1266. The resulting strains exhibited higher feather degradation efficiency with an improved rate of 15% over the wild-type strain (de Paiva et al., 2018).

### Keratinases

**Figure 3:**
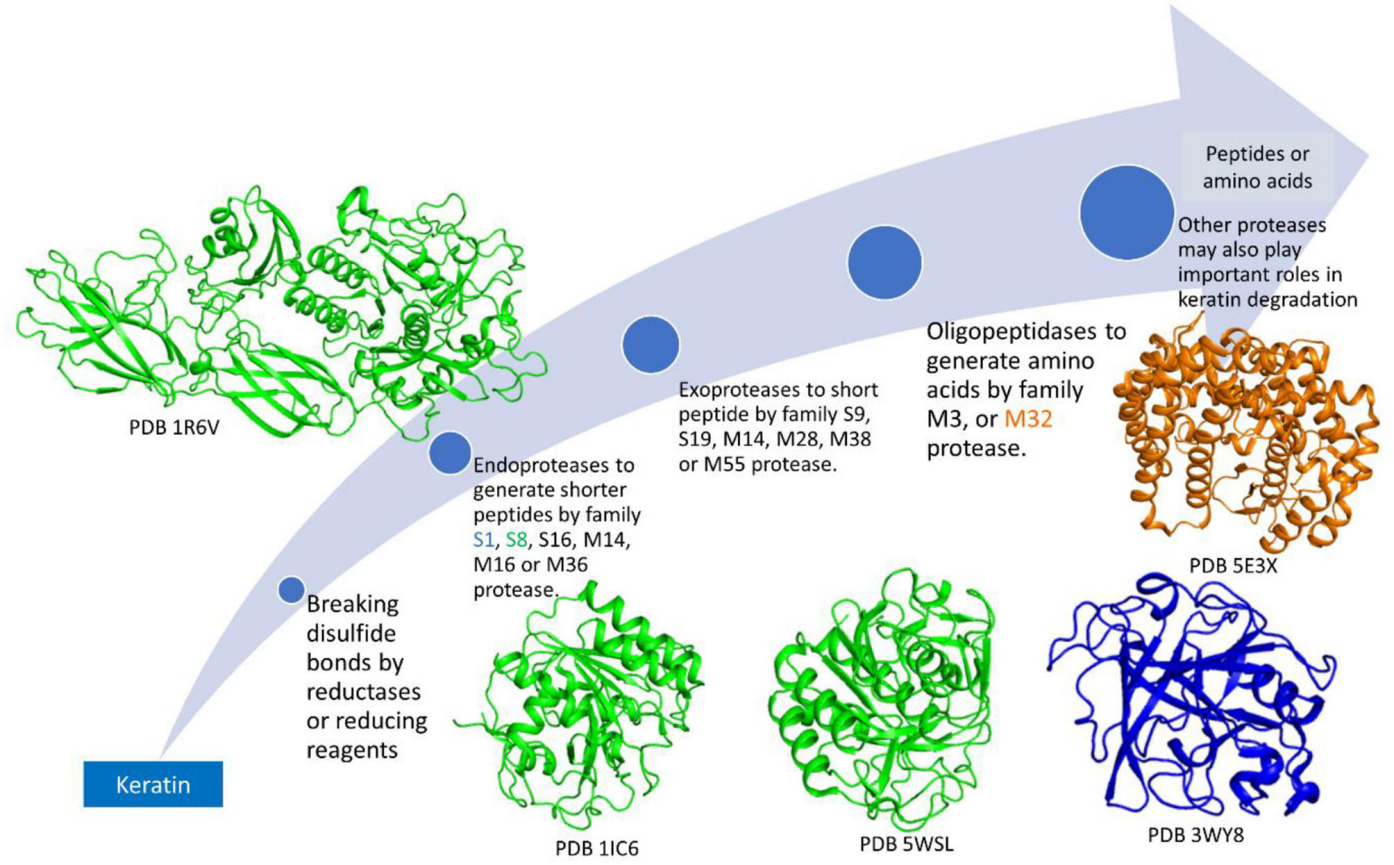
Microbial enzymes involved in keratin degradation. There are at least 14 different protease families identified in microorganisms. These proteases play different roles in keratin degradation. Keratinases among the same family may have different structures. The structures from protein databank (PDB) are shown with the access numbers indicated in the figure.

Due to the insoluble property of keratin, enzymes secreted outside of the cells will play a major role in degradation (Williams and Shih, 1989; da Silva, 2018; Tamreihao et al., 2019; de Menezes et al., 2021). Because of the stable structure of keratin, normal proteases cannot degrade the substrate (Kasperova et al., 2013). Therefore, the degradation of keratin should include breaking the disulfide bonds followed by peptide degradation by proteases (Ramnani et al., 2005; Korniłłowicz-Kowalska and Bohacz, 2011). Although keratin degradation is a complicated step, these two steps including disulfide bond breakage by enzymes or reducing reagents and proteolysis of the peptides are indispensable. Other mechanisms such as mechanical destruction by fungi may also be included in keratin degradation (Korniłłowicz-Kowalska and Bohacz, 2011). As the disulfide bonds link polypeptides to form fibrous structures, breaking the disulfide bonds may be the first step in microbial degradation (Kasperova et al., 2013; Lange et al., 2016; Mercer and Stewart, 2018; Flückger et al., 2020). This step can be catalyzed by the produced inorganic sulfite and disulfide reductase (Korniłłowicz-Kowalska and Bohacz, 2011). Some disulfide reductases involved in keratin degradation have been isolated from microorganisms and characterized (Kasperova et al., 2013; Navone and Speight, 2018). Keratinases are another component for breaking the poly-peptides into shorter peptides and amino acids (Qiu et al., 2020). As a protease usually prefers to a cleavage site formed by specific residues, different types of proteases are needed to degrade keratin into amino acids completely (Qiu et al., 2020). Diverse proteases from different microorganisms have been identified with the molecular weights at the range from 20 to 130 kDa (Kanoksilapatham and Intagun, 2017; Sharma and Devi, 2018; Pinski et al., 2020; Qiu et al., 2020) and preference to various pHs, temperatures, and substrates (Tatineni et al., 2007; Tiwary and Gupta, 2010b; Rai and Mukherjee, 2011; Khodayari and Kafilzadeh, 2018; Arokiyaraj et al., 2019; Nnolim et al., 2020a; Almahasheer et al., 2022). Even the structures of the keratinases within the same protease family exhibit different folding and contain different structural elements (Figure 3).

Recombinant keratinases can be obtained when the sequences are available while the selection of host cells and expression systems are critical because the target protein may not be easily overexpressed (Descamps et al., 2003; liu et al., 2013b; Fang et al., 2014; Yong et al., 2020; Parinayawanich et al., 2021; Yahaya et al., 2021). This method is very useful to obtain keratinases with high purify and study the enzymes from pathogenic microorganisms (Muhammed et al., 2021). Enzymatic characterization and mutagenesis can be readily studied using recombinant proteins (Zhang et al., 2020). Recombinant bacterial keratinases can be produced in *Escherichia coli* (*E. coli*) (Tiwary and Gupta, 2010a). Interestingly, the yield of the recombinant keratinase in expression systems such as *E. coli* may not be higher than that from its native source. To increase the yield of recombinant protein multiple copies of kerA gene were inserted into the chromosome of expression bacterial cells (Wang et al., 2004). The amino acid sequences of most keratinases are available, and the structures of several keratinases have been determined, which makes it possible to perform rational protein design for improving the activity and thermal stability of keratinases. Indeed, site-directed mutagenesis (N122Y, N217S, A193P, and N160C) was used to improve the keratinase of *Bacillus licheniformis* BBE11 (liu et al., 2013a). It is shown that point mutation N122Y in keratinase increased the enzymatic activity with an approximately 5.6-fold (liu et al., 2013a). As the N- and C-termini of a protease may play a regulatory role in the enzymatic activity, swapping these regions with similar domains of enzymes from other bacteria can also result in an enzyme with improved activity and stability (Fang et al., 2016; Peng et al., 2021).

Extensive studies have been performed to explore the strains, enzymes, and conditions required for the degradation of keratin and keratin-containing wastes such as feathers (Onifade et al., 1998; Gupta and Ramnani, 2006; Brandelli et al., 2010; Chaturvedi et al., 2014; Sahni et al., 2015; Sharma and Gupta, 2016; Vidmar and Vodovnik, 2018). It has been noted that most studies have been focused on a single strain, one enzyme using one substrate in the assay, and one processing condition. Keratin degradation is a complicated step that requires multiple strains or multiple enzymes in nature. A more comprehensive analytical strategy is needed to achieve an efficient degradation. Bacterial consortia might be of great interest to process keratin-containing wastes efficiently while more careful studies are needed to identify microorganisms required. Due to the amount of keratin in nature, it is not surprising that microbial keratinases would have industrial and biotechnological applications (Table 1) (Onifade et al., 1998; Gupta and Ramnani, 2006; Rai et al., 2009; Syed et al., 2009; Brandelli et al., 2010; Sahni et al., 2015; Kanoksilapatham and Intagun, 2017; Gegeckas et al., 2018; Vidmar and Vodovnik, 2018). Keratin is rich in carbon, nitrogen, and sulfur which can be converted into diverse products (McKittrick et al., 2012; Wang et al., 2016). Modern agriculture requires certain types of fertilizers that can recover the loss of nutrients in soil and sustain the microbial ecosystem for the growth of plants and fixing/solubilizing nutrients in soil. Keratin-containing wastes such as feathers and its degradation bacteria will be a source for producing biofertilizers with a combination of the merits from both organic fertilizer and biofertilizers for sustainable agriculture.

**Table 1:** Composition of the microbial feather hydrolysate.

**Components**	**Remarks**	**References**
Amino acids	Over 15 amino acids can be released from feathers. The composition of amino acids is variant among different feathers.	Saravanan and Dhurai, 2012; Tamreihao et al., 2019
Indole-3-acetic acid (IAA)	It is not a component of keratin. IAA can be produced using tryptophan as a precursor and it is important for plant growth.	Tamreihao et al., 2017
Peptides	Keratin degradation is a complicated step. Peptides with different length can be produced during degradation.	Qiu et al., 2020
Microorganisms	Microorganisms used in feather degradation will be in the feather hydrolysate. Their cells can serve as nutrient for plant or animals.	Gurav et al., 2020; Nnolim et al., 2020b; Qiu et al., 2020
Enzymes	Keratinases can be produced during feather degradation. They will be one of the components in the feather hydrolysate.	Gurav et al., 2020; Nnolim et al., 2020b; Qiu et al., 2020
Others	Many other microbial products during bacterial growth can be released. These products include proteins, lipids or other components such as phosphate and sulfur.	Gupta and Ramnani, 2006; Brandelli et al., 2010; Bhange et al., 2016; Lange et al., 2016

## Converting Keratin to Biofertilizers

**Figure 4:**
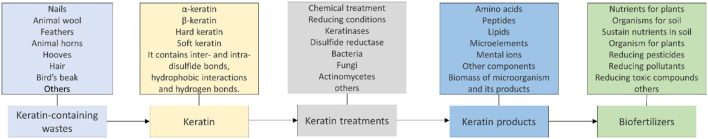
Keratin and its potential to be converted into biofertilizers. The source of keratin and its conversion to biofertilizers are listed. Keratin-containing wastes, structures of keratin, methods for keratin treatment, keratin products, and application of biofertilizers are indicated.

Keratin degrading microorganisms and the related enzymes have been summarized (Adav et al., 2018; Li, 2019, 2021; Hassan et al., 2020a; Qiu et al., 2020). Keratin and keratin-containing wastes such as feathers are a valuable source for carbon, nitrogen, and sulfur because of the components of polypeptides in the wastes (Pettett et al., 2004; Gurav and Jadhav, 2013; Qiu et al., 2020) (Figure 4). During the growth of microorganisms, other nutrients such as phosphate will be taken from the medium for energy synthesis and building up cell organelles such as cell membrane. Keratin-containing wastes will play a role in sustainable agriculture by providing value-added products (da Silva, 2018; Shestakova et al., 2021). The keratin hydrolysate influences the plant growth because of the peptides and amino acids which play versatile roles in plants (Bhari et al., 2021). These products have been shown to have an impact on the stimulation of carbon and nitrogen metabolism, which may result in the difference in seeds germination, seedling growth, and activation of the plant proton pump (Calin et al., 2019). Peptides in the keratin hydrolysate may exhibit hormonal activities. These products can also play a role in stimulating plant growth through their indirect effect on nutrient update and usage (Raguraj et al., 2022). The amino acids in keratin hydrolysate will have an impact on the rhizospheric microbes by serving as a nitrogen source, which in turn will affect the action of the microorganism on the root of the plant (Moe, 2013). Therefore, hydrolysate derived from keratin wastes is a promising biofertilizer. Studies have shown that microbial keratin hydrolysate can serve as a biofertilizer to promote plant growth (Table 2). Addition of hydrolysate such as 5 g feather in 250 g soil and 1 kg in 10 kg soil enhanced plant growth (Kumari and Kumar, 2020). Biofertilizers derived from keratin-containing wastes can be obtained through the following mechanisms. First, the polyproteins in keratin can be converted into amino acids to serve as nitrogen sources for plant growth (Geisseler et al., 2021). Second, the microorganisms used in waste treatment will be another source of nutrients for the plant growth. The microbial products of feathers can be used as a fertilizer to improve the growth of plants (Gurav et al., 2020; Ortiz and Sansinenea, 2021). Third, the microorganisms can also play a role in absorbing the excess inorganic nutrient in soil released from synthesized fertilizers to prevent nutrient loss (Maitra et al., 2022). Lastly, mixing other microorganisms used in biofertilizers with microbial-treated keratin wastes will result in a new fertilizer with the properties of both organic fertilizers and biofertilizers (Mitter et al., 2019). An ideal microorganism is the one used in biofertilizers with the capability to degrade keratins.

**Table 2:** Some examples of microbial feather hydrolysate as fertilizer to promote plant growth.

**Microorganisms**	**Feather/soil**	**Test plant**	**References**
*Amycolatopsis* sp.	NA	Rice	Tamreihao et al., 2017
*Aspergillus niger*	1–3 kg/10 kg	Cowpea	Adetunji et al., 2012
*Alternaria tenuissima*	2–5 g/250 g	Chickpea	Kumari and Kumar, 2020
*Bacillus aerius*	10–30 ml	Mung beans	Kaur et al., 2021
*Bacillus amyloliquefaciens*	153 ml/ kg	Mung seeds	Bose et al., 2014
*Bacillus cereus*	NA	Rice seeds	Sivakumar et al., 2012
*Bacillus pumilus*	188 ml/kg	Carrot and cabbage	Kim et al., 2005
*Bacillus* sp.	180 mg N/ kg	Lettuce	Sobucki et al., 2019
*Bacillus subtilis*	NA	Mung beans	Bhange et al., 2016
*Cyberlindnera fabianii*	NA	Pea	Abdel-fattah et al., 2015
*Chrysosporium tropicum*	5 g/200 g	Pea and rice	Kumar et al., 2017
*Chryseobacterium* sp.	NA	Banana	Gurav and Jadhav, 2013
*Paenibacillus woosongenesis*	10–30 ml /100 g	Bengal gram	Paul et al., 2013
*Streptomyces sampsonii*	0.5–1.5 ml/100 g	Wheat	Jain et al., 2016

Unlike organic and inorganic fertilizers rich in nutrients, biofertilizers contain living microbes that play important roles in promoting the growth of crops through enlarging the supply of essential nutrients absorbable by the crops (Jiménez-Mejía et al., 2022; Maitra et al., 2022). These living organisms that are helpful for plant growth usually include bacteria, blue-green algae, and mycorrhizal fungi (Kumar et al., 2022b) and many types of biofertilizers are commercially available. These organisms can help extracting minerals from organic materials or turning nutrients from one type of forms to another such as nitrogen fixation (Pirttilä et al., 2021; Kumar et al., 2022b) (Figure 4). The following types of microorganisms are commonly used in biofertilizers. Rhizobium is one of the symbiotic nitrogen-fixing bacteria used in biofertilizers and able to fix nitrogen for growth of the plants (Gopalakrishnan et al., 2015; Mendoza-Suárez et al., 2021). Azospirillum live at the root of the plants serving as nitrogen-fixing bacteria without developing an intimate relationship with plants (Berg, 2009). Some organisms such as cyanobacteria have a symbiotic association with the plants can also be used in biofertilizers (Bhattacharjee et al., 2008; Bononi et al., 2020). Due to the association of this type of organisms with plants, potential challenges may occur during the preparation or storage of the biofertilizers. Other bacteria whose growth and living are not relying on the plants such as free-living nitrogen-fixing bacteria are of great interest in keratin-derived biofertilizers (Lucy et al., 2004; Deka et al., 2020).

The organisms used in keratin degradation have different mechanisms of action from those applied in biofertilizers (Korniłłowicz-Kowalska and Bohacz, 2011; Ortiz and Sansinenea, 2021; Sypka et al., 2021). Theoretically, the presence of microbial keratin hydrolysate in soil will play a role in the ecosystem because the growth of microorganisms also results in nitrogen-fixing and absorption of phosphate and potassium from the soil (Kumar et al., 2022b). Microbial keratin hydrolysate containing mixtures of microorganisms and products can be considered as a form of biofertilizers because of the presence of living organisms and nutrients. To achieve higher benefit to the ecosystem, the following strategies will be useful. First, the selection of suitable keratin-degrading organisms in wastes treatment should be considered. Some microorganisms such as *Bacillus subtilis* can degrade keratin and play a role in phosphate solubilization to enhance plant nutrient uptake (Santoyo et al., 2012; Jeevana Lakshmi et al., 2013; Chaturvedi et al., 2014; Gupta et al., 2017; de Paiva et al., 2018; Kumar et al., 2022b). This type of organisms will be the ideal candidate for converting keratin-containing wastes into biofertilizers. To obtain such organisms, samples used for strain isolation of keratin degrading microorganisms should be obtained from soil or other environment where the organisms beneficial for growth of plant can survive (Jagadeesan et al., 2020). Second, mixing multiple organisms during the processing of keratin-containing wastes may improve the effect of biofertilizer on plant growth (Zahra et al., 2020). Two classes of organisms are important. One class of organisms can degrade keratin and the other will be beneficial for plant growth and the soil ecosystem. The later class of organisms can be mixed with microbial keratin hydrolysate to generate a fertilizer with the function of both organic and biofertilizers (Sabir et al., 2021). Lastly, in the microbial treatment of keratin-containing wastes such as feathers, mixing the processed wastes with other wastes such as cellulose and organisms can be considered (Brandelli et al., 2015; Bohacz, 2019; Patinvoh et al., 2020), which might provide a more complicated system for both plant and organism growth.

## Perspectives

With the development of economics and the increment of global population, the demand for food and meat such as chicken is increasing. Feathers production from the poultry industry is growing rapidly and this keratin-containing waste can be a threat to the environment and cause a waste of carbon and nitrogen sources if it is not treated properly (Pahua-Ramos et al., 2017; Kowalczyk et al., 2018). The quality of soil needs to be sustained when the quality and quantity of food production are enhanced in modern agriculture. To maintain sustainable agriculture, it is essential to reduce the usage of inorganic fertilizers and balance the ecosystem in soil by application of new fertilizers (Young and Ritz, 2000; Orr et al., 2011; Liu et al., 2021). Organic fertilizers and biofertilizers are indispensable for sustainable agriculture (Hernández-Fernández et al., 2021; Wang et al., 2021; Kumar et al., 2022a). Converting keratin-containing wastes into biofertilizers can overcome some disadvantages of organic fertilizers such as toxicity to plants and soil under certain conditions, and low nutrient content of organic mulches.

**Figure 5:**
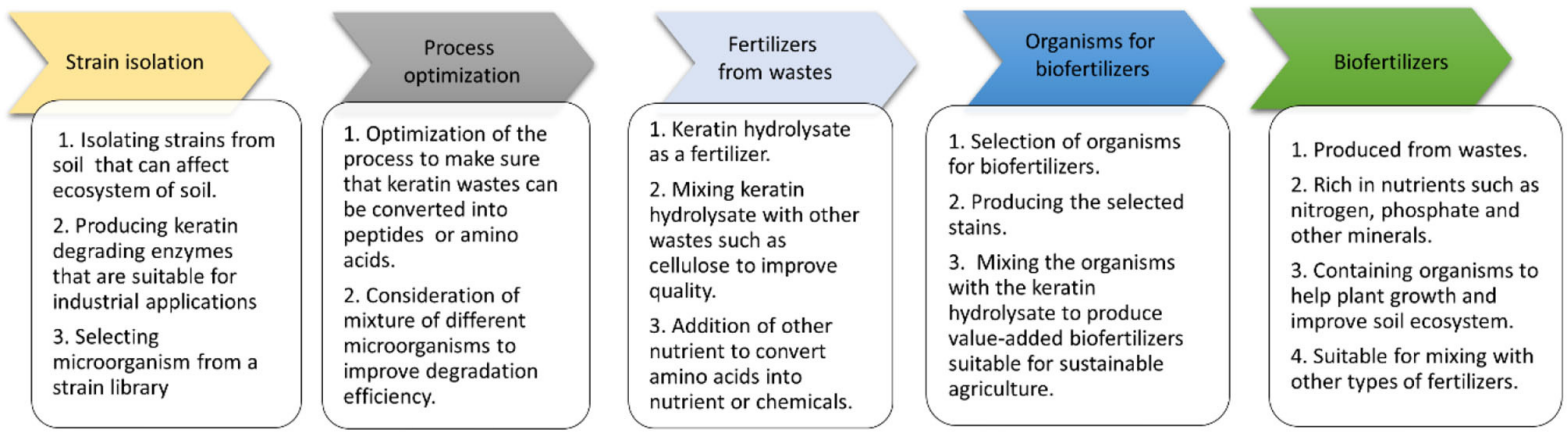
A strategy to convert keratin-containing waste into biofertilizers.

To produce biofertilizers using keratin-containing materials, microbial treatment of keratins is feasible (Tamreihao et al., 2019; Nnolim et al., 2020b; Izydorczyk et al., 2022). The strains used in treatment need to be identified or selected from strain libraries. The selection of microbial consortia is of great interest while more studies are still needed to identify or build up a suitable system for keratin degradation. Breaking the disulfide bonds in keratin to release the polypeptides is a critical step to release soluble proteins that can be readily used by many organisms (Gurung et al., 2018; Sharma and Devi, 2018; Qiu et al., 2020; Nnolim and Nwodo, 2021). Studies on the detailed components involved in keratin degradation are still required. With the development of analytical methods such as mass spectrometry and wide application of genome sequencing, the degradation of keratin can be analyzed in more details (Khodayari and Kafilzadeh, 2018; Navone and Speight, 2018; Fang et al., 2019; Hamiche et al., 2019; Hassan et al., 2020b; Yahaya et al., 2021). The addition of organisms suitable for the growth of plants to keratin degradation product will result in a high-quality biofertilizer to provide nutrients for the growth of plant, sustain the microorganism in the soil and reduce the usage of pesticides (Figure 5). The organisms used in conventional biofertilizers can be screened from the soil or purchased from commercial resources, which can be applied in keratin treatment or mixed with microbial-treated keratins to produce biofertilizers with multiple functions to contribute to the sustainable agriculture (Bano et al., 2021; Kumar et al., 2021; Lee et al., 2021; Rios-Galicia et al., 2021; Seenivasagan and Babalola, 2021).

In summary, keratin-containing waste is rich in amino acids serving as a valuable resource for the growth of plants. With proper treatment, wastes such as feathers can be converted into biofertilizers to play a role in sustainable agriculture. With a proper microbial treatment of feathers and the addition of other organisms, feathers will be converted into biofertilizers applied to sustainable agriculture.

## Author Contributions

The author confirms being the sole contributor of this work and has approved it for publication.

## Funding

This research was supported by funds from the Hundred-Talent Program (Grant Numbers: 2020GDASYL-20200102010 and 2020GDASYL-20200102009) and GDAS' Project of Science and Technology Development (2022GDASZH-2022010110), Guangdong Academy of Sciences, China.

## Conflict of Interest

The author declares that the research was conducted in the absence of any commercial or financial relationships that could be construed as a potential conflict of interest.

## Publisher's Note

All claims expressed in this article are solely those of the authors and do not necessarily represent those of their affiliated organizations, or those of the publisher, the editors and the reviewers. Any product that may be evaluated in this article, or claim that may be made by its manufacturer, is not guaranteed or endorsed by the publisher.
